# Prediction of Physical Frailty in Orthogeriatric Patients Using Sensor Insole–Based Gait Analysis and Machine Learning Algorithms: Cross-sectional Study

**DOI:** 10.2196/32724

**Published:** 2022-01-05

**Authors:** Moritz Kraus, Maximilian Michael Saller, Sebastian Felix Baumbach, Carl Neuerburg, Ulla Cordula Stumpf, Wolfgang Böcker, Alexander Martin Keppler

**Affiliations:** 1 Department of Orthopaedics and Trauma Surgery Musculoskeletal University Center Munich Ludwig-Maximilians Universität Munich Munich Germany

**Keywords:** wearables, insole sensors, orthogeriatric, artificial intelligence, prediction models, machine learning, gait analysis, digital sensors, digital health, aging, prediction algorithms, geriatric, mobile health, mobile insoles

## Abstract

**Background:**

Assessment of the physical frailty of older patients is of great importance in many medical disciplines to be able to implement individualized therapies. For physical tests, time is usually used as the only objective measure. To record other objective factors, modern wearables offer great potential for generating valid data and integrating the data into medical decision-making.

**Objective:**

The aim of this study was to compare the predictive value of insole data, which were collected during the Timed-Up-and-Go (TUG) test, to the benchmark standard questionnaire for sarcopenia (SARC-F: strength, assistance with walking, rising from a chair, climbing stairs, and falls) and physical assessment (TUG test) for evaluating physical frailty, defined by the Short Physical Performance Battery (SPPB), using machine learning algorithms.

**Methods:**

This cross-sectional study included patients aged >60 years with independent ambulation and no mental or neurological impairment. A comprehensive set of parameters associated with physical frailty were assessed, including body composition, questionnaires (European Quality of Life 5-dimension [EQ 5D 5L], SARC-F), and physical performance tests (SPPB, TUG), along with digital sensor insole gait parameters collected during the TUG test. Physical frailty was defined as an SPPB score≤8. Advanced statistics, including random forest (RF) feature selection and machine learning algorithms (K-nearest neighbor [KNN] and RF) were used to compare the diagnostic value of these parameters to identify patients with physical frailty.

**Results:**

Classified by the SPPB, 23 of the 57 eligible patients were defined as having physical frailty. Several gait parameters were significantly different between the two groups (with and without physical frailty). The area under the receiver operating characteristic curve (AUROC) of the TUG test was superior to that of the SARC-F (0.862 vs 0.639). The recursive feature elimination algorithm identified 9 parameters, 8 of which were digital insole gait parameters. Both the KNN and RF algorithms trained with these parameters resulted in excellent results (AUROC of 0.801 and 0.919, respectively).

**Conclusions:**

A gait analysis based on machine learning algorithms using sensor soles is superior to the SARC-F and the TUG test to identify physical frailty in orthogeriatric patients.

## Introduction

The physiological process of aging is inevitably connected to a decrease in physical performance [1]. It has been estimated that approximately 30% of the US population above the age of 55 years suffer from moderate to severe physical limitations [2]. In an orthogeriatric patient population, the assessment of physical frailty is of particular importance, as it is not only strongly associated with falls but also to an inferior outcome following surgery [3]. Consequently, it is of upmost importance to test for and thereby objectify physical impairment (ie, frailty).

Various individual parameters have been proposed to assess physical performance, including handgrip strength, daily step count, and gait speed. However, all of these have considerable interindividual variation [4]. Along with individual physiologic parameters, a variety of questionnaires such as the Barthel index [5], De-Morton Mobility index [6], or FRAIL scale [7] have been developed to quantify frailty. However, these questionnaires have proven to be inferior to the more complex physical assessments [8]. The Short Physical Performance Battery (SPPB) [9] is often considered one of the benchmark tests to assess frailty [8]. The SPPB combines multiple physical assessments, including gait, balance, and strength [10]. There is a consensus that screening for physical frailty is not only the prerequisite for successful individual patient care but also for cost-effectiveness [11]. Nonetheless, an international consensus on the most appropriate screening method is still missing [12].

As outlined above, comprehensive physical stance and gait assessments might be the most effective approach to quantify frailty. A new approach to assess physical activity and gait parameters includes the use of wearables and physical activity monitors [13]. These devices enable physicians and researchers to assess physical activity comprehensively under real-life conditions, and they have already been successfully applied to assist in the diagnosis of musculoskeletal diseases and to monitor rehabilitation [14-17]. A more recent development is sensor insoles with pressure and gyroscope sensors. These insoles can be easily inserted into any shoe and allow for the assessment of several gait parameters in an outpatient setting and also during various daily activities. This might provide a more feasible alternative to time-consuming assessments in specialized gait laboratories.

Although sensor insoles might help in the assessment of frailty, the large number of data points generated necessitates advanced statistical analysis. The random forest (RF) based on decision trees or the K-nearest neighbor (KNN) based on the Euclidean distance between points in high-dimensional space are two suitable strategies to develop clinical decision algorithms [18].

The aim of this study was to compare the classification capability of insole data collected during the Timed-Up-and-Go (TUG) test—a clinical gait test to assess a patient’s mobility and risk of falling—to SARC-F (a five-item questionnaire for the quick assessment of the risk of sarcopenia, assessing strength, assistance with walking, rising from a chair, climbing stairs, and falls) and the TUG test to assess physical frailty, defined by the SPPB, using machine learning algorithms.

## Methods

### Patient Selection

Patients presenting to our orthogeriatric outpatient clinic for an osteoporosis diagnosis or therapy between December 2020 and March 2021 were invited to participate in this study. Inclusion criteria were aged >60 years, independent ambulation without any walking aids, and no mental or neurological impairment. Patients were informed of the study details, including the anonymized evaluation of the collected data, and then provided written consent. This cross-sectional study was approved by the local ethics committee (#19-177).

### General Data Assessment

All data were collected in a standardized fashion by a unique, specially trained investigator. Demographic data included age, weight, height, BMI, body composition, general health-related quality of life assessed by the European Quality of Life 5-dimension (EQ-5D-5L) questionnaire [19], and the sarcopenia and physical frailty screening questionnaire SARC-F [20]. All questionnaires were completed together with the patients to obtain the highest possible data quality. Body composition (ie, body fat and muscle percentages) was measured using a clinically validated body composition monitor (BF511, Omron-Healthcare, Kyoto, Japan).

### Assessment of Physical Frailty

Physical frailty was assessed by three different means: the SPPB, the TUG test, and digital insole gait parameters assessed during the TUG test using sensor insoles (Science3, Moticon, Munich, Germany).

The SPPB [9] is considered the benchmark test to assess physical frailty and was therefore used as the primary outcome parameter [8]. The SPPB is comprised of multiple tests for gait and stance safety, as well as lower-extremity strength and performance [10]. This tool scores the ability to stand in three different positions for 10 seconds, the time required to walk 3 meters, and the time it takes to rise from and sit down on a chair 5 times. Points are awarded for each subtest according to the time achieved, with a maximum score of 12 and a minimum score of 0. Patients with SPPB scores≤8 are considered to be physically frail [21,22]. The binary SPPB score (not physically frail vs physically frail) was used as the classification label for the machine learning models applied in this study.

The TUG test measures the time a patient takes to rise from a chair (height 46 centimeters), walk 3 meters, turn 180 degrees, and return to their initial seating position [23]. A duration of 12 seconds or longer has been associated with a higher probability of physical frailty [24]. Therefore, a cut-off value of 12 seconds was chosen to classify patients into physically frail and not physically frail groups.

The gait parameters were assessed by Science3 digital sensor insoles during the TUG test. Each of these insoles has 19 pressure sensors and a 3D gyroscope sensor to measure a variety of temporal, spatial, and local gait parameters, including gait speed and pressure distribution [25,26]. The parameters assessed are outlined in detail in Table 1.

**Table 1:** Overview of all insole gait parameters assessed.

Parameter	Unit
TUG^a^ test time	seconds
Steps	number
Mean length of gait line	millimeters
Standard deviation x/y of gait line	meters
Mean total force during stance	Newtons
Mean gait cycle time	seconds
Mean gait cadence	strides/minute
Mean double support time	seconds
Mean acceleration over gait cycle (x/y/z)	*g*
Mean stride length	meters
Mean fraction of stance phase	%
Mean fraction of swing phase	%
Walking distance	meters
Mean walking speed	meters/second
COP^b^ variability (left/right)	meters
COP trace length (left/right)	meters

^a^TUG: Timed-Up-and-Go.

^b^COP: center of pressure.

### General Statistical Analysis

Unpaired *t* tests were used with *α* adjustment according to the Benjamini and Hochberg method [27] to compare interval-scaled, normally distributed variables (demographics, questionnaires, and gait parameters) between patients with and without physical frailty. Data are expressed as mean (SD). The effect size is expressed as the standardized mean difference.

### Prediction Algorithms

To train the prediction algorithms, all collected performance- and nonperformance-related variables were used to train a recursive feature elimination algorithm that can identify the most relevant parameters for distinguishing patients with (SPPB score≤8) and without (SPPB score>8) physical frailty. For this purpose, the feature elimination algorithm was used to choose the best suitable variables based on an RF algorithm from the ranger package [28]. Gini impurity was used to rank the variables in order of their importance, as this measure is particularly suited to assess how well certain variables divide up a data set [29]. Based on this ordering of the variables, the variables were gradually removed until the lowest possible classification error was achieved. The classification error was chosen as the performance measure for the recursive feature selection, since the main focus was on maximizing the accuracy of the models developed later.

Two supervised machine learning algorithms, KNN [30] and RF, were used for further analysis using the previously selected variables. Both algorithms rely on being trained with labeled training data with a subsequent performance evaluation using test data. Prior to the training and tuning processes, the data were split into a training and a testing data set at a 70:30 ratio. The training process included an internal 3-fold cross-validation step. As hyperparameter tuning is essential for supervised machine learning algorithms to increase the accuracy of the classification [31], both algorithms were subjected to a tuning process that optimizes all variables to be tuned simultaneously, exclusively using the training data set. For the KNN, the tuning range for the number of neighbors was set from 1 to 22. For the type of kernels, the four variants rectangular, Gaussian, rank, and optimal were tested. For the unit of measurement of the distance, the options Euclidean distance, absolute distance, and Minkowski distance were available. For the RF, the number of variables considered as split candidates within a tree was tuned in the range of 1 to 7, the maximum number of branches in a tree was in the range of 2 to 10, and the number of trees in the RF was set from 100 to 1000. The nested resampling technique was used to enable better estimation of the true model performance on unseen data [32]. The 30% of the data not seen by the model were used to compare the performance of the different models subsequently.

To compare the generated algorithms to the classification properties of the TUG and SARC-F, confusion matrices and receiver operating characteristic (ROC) curves were created based on a logistic regression for the SARC-F using solely the score achieved and for the TUG using only the time taken to complete the test so as to compare the different prediction strategies. All data were collected in a REDCap study database [33] and analyzed in a standardized manner with RStudio software (version 1.3.1093), R (version 4.0.3), using the packages dplyr (version 1.0.2), Hmisc (version 4.6-0), ggplot2 (version 3.3.2), caret (version 6.0-86), and mlr3 (version 6.0-86) [34]. The code used to create and compare the models to the established tests has been made publicly available on GitHub [35].

## Results

All of the 57 eligible consecutive orthogeriatric patients were included in the final analysis. The patients’ mean age was 77 (SD 6) years and 93% were women. Classified by the SPPB, 23 patients (40%) had physical frailty. Table 2 shows the comparison of all assessed general parameters between the patients with and without physical frailty. Only age, EQ-5D-5L index, and SARC-F score differed significantly between the two groups. It should be emphasized that the average age of the patients with physical frailty was more than 5 years above the average age of the patients without physical frailty. In parallel, the mean health index of the patients with physical frailty determined by the EQ-5D-5L was almost 0.2 points below that of the patients without physical frailty. All other collected demographic data such as weight, height, BMI, body fat, and muscle mass did not differ significantly between the two groups.

The between-groups comparison of the digital gait analysis is presented in Table 3. The two groups differed significantly for all insole-generated gait parameters (all *P*<.05).

**Figure 1:**
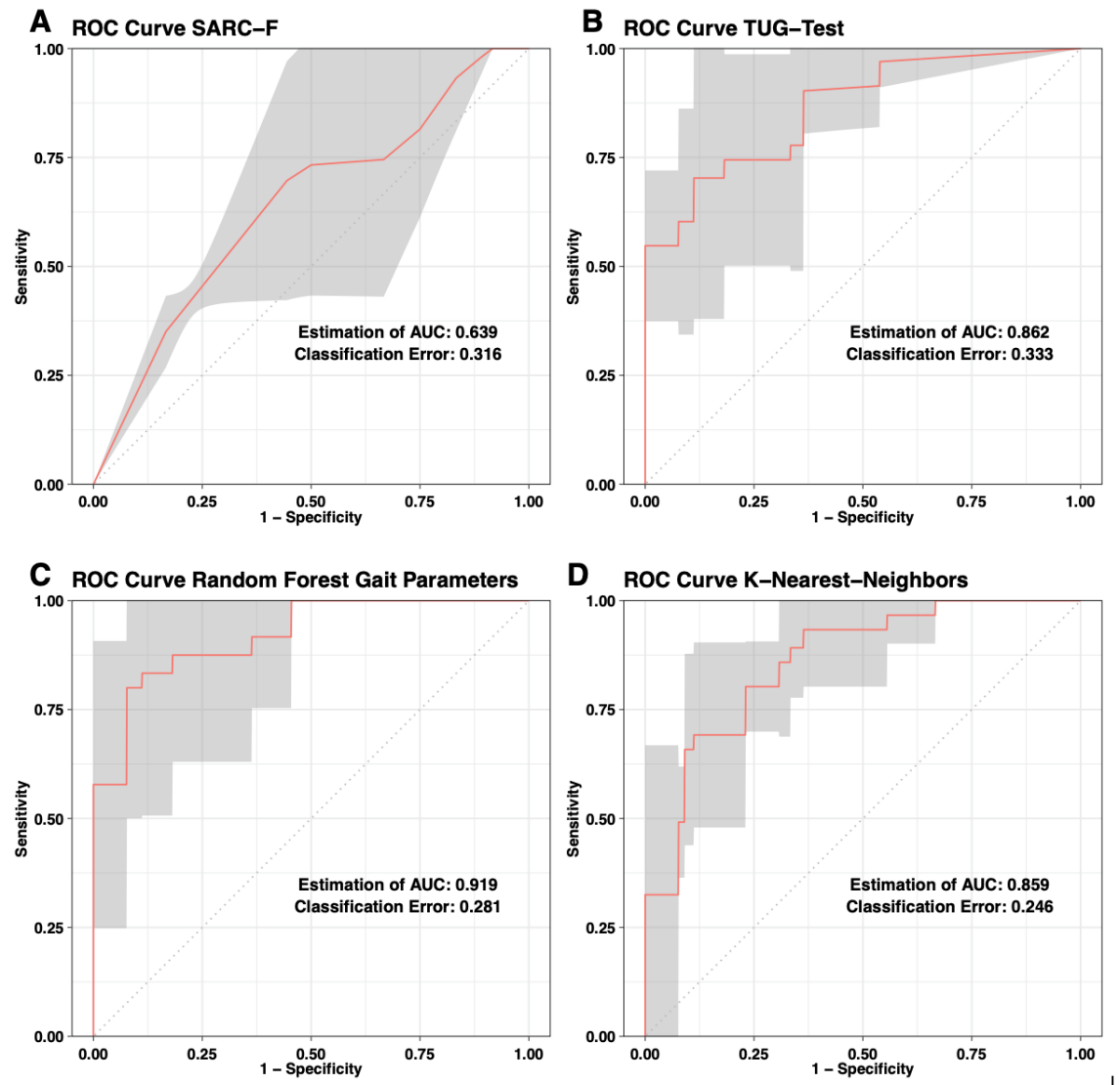
Comparison of the receiver operating characteristic (ROC) curves of the classification properties of the sarcopenia index SARC-F (A), Timed-Up-and-Go (TUG) test (B), and the random forest (C) and k-nearest neighbor (D) algorithms. AUC: area under the ROC curve.

The classification errors of the TUG test and SARC-F to identify patients with physical frailty were 0.333 and 0.316, respectively. However, the area under the ROC curve (AUROC) for the TUG test was higher when compared with that of the SARC-F (0.862 vs 0.639; Figure 1A, Figure 1B).

The RF-based recursive feature elimination algorithm was trained to extract the most important features for classifying physical frailty using all parameters collected, except the SPPB, TUG test, and SARC-F, as they either define the result or represent the classification methods to be compared.

**Figure 2:**
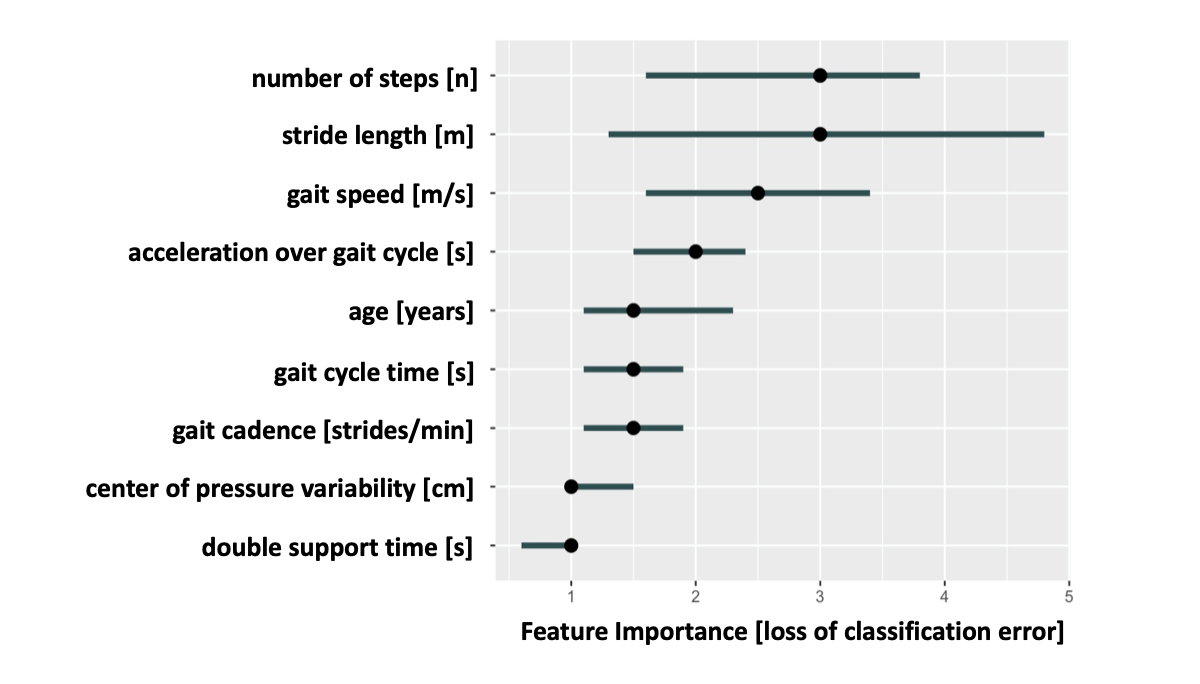
Selected parameters based on the recursive feature elimination algorithm, ordered by their importance for reduction of classification error ranked by Gini-Impurity [29].

Based on the defined criteria, the 9 parameters outlined in Figure 2 were included. Notably, 8 out of the 9 parameters selected were gait parameters collected by the insoles (Figure 2). The number of steps and the step length were the most decisive factors for the identification of physical frailty by the algorithm. The gait speed followed in third place. Of the variables selected, double support seemed to have the least effect on classification.

These variables were then used to train the two classification algorithms KNN and RF. The tuning process resulted in an optimal combination of hyperparameters for the KNN as follows: k=15, a “rank” kernel, and the Minkowski distance. The optimal combination for the RF was 7 split variables, 6 branches, and 550 trees.

To compare the classification abilities of the TUG and the SARC-F with the algorithms created, a logistic regression was carried out on the SARC-F score and the TUG time on the dependent variable physical frailty and the ROC curve was drawn (Figure 1A-D). Table 4 summarizes the prediction accuracy of the four classifiers. Both classical approaches were outperformed by the machine learning–based models in terms of classification error (KNN=0.246, Figure 1D; RF=0.281, Figure 1C). The AUROC for the RF was slightly superior to that of the KNN (Table 4). Overall, the KNN showed the lowest error rate in classification at 24.6% (Figure 1). RF showed the largest AUROC value and thus appears to be the most suitable for classification. In the conventional tests, the TUG test was far superior to the SARC-F in terms of area under the ROC curve and classification error. The KNN showed the lowest classification error rate, but had a slightly smaller AUROC value than those of the RF and the TUG test.

**Table 2:** Comparison of demographics, body composition, physical activity, physical performance, and health questionnaire scores between patients with and without physical frailty.

Variable		No physical frailty (n=34)	Physical frailty (n=23)	*P* value	SMD^a^
Age (years), mean (SD)		74.76 (5.92)	80.00 (5.82)	.002	0.892
BMI (kg/m^2^), mean (SD)		24.42 (4.81)	24.66 (3.79)	.84	0.055
Height (cm), mean (SD)		160.94 (6.37)	160.56 (7.84)	.85	0.053
Weight (kg), mean (SD)		62.77 (9.72)	63.45 (9.61)	.80	0.070
Body fat (%), mean (SD)		30.15 (8.55)	32.14 (7.86)	.37	0.243
Visceral fat (%), mean (SD)		7.95 (3.21)	8.71 (2.72)	.34	0.254
Muscle mass (%), mean (SD)		30.26 (4.20)	28.52 (3.29)	.09	0.460
Resting metabolism (kcal), mean (SD)		1345.32 (110.40)	1341.29 (123.22)	.90	0.034
Calf circumference, mean (SD)		35.04 (3.12)	34.31 (3.30)	.41	0.228
EQ-5D-5L^b^ index, mean (SD)		0.84 (0.16)	0.65 (0.27)	.007	0.818
SPPB^c^ score (points), mean (SD)		11.30 (0.79)	6.44 (2.06)	<.001	–3.106
SPPB score≤8, n (%)		0 (0)	23 (40)	<.001	
**SARC-F^d^ score, n (%)**				.01	1.002
	0	22 (65)	6 (26)		
	1	8 (24)	7 (30)		
	2	2 (6)	3 (13)		
	3	0 (0)	4 (17)		
	4	2 (6)	3 (13)		
**Number of falls in past year, n (%)**				.31	0.422
	0	24 (71)	12 (52)		
	1-3	7 (21)	9 (39)		
	>3	3 (9)	2 (9)		
BMD^e^ femoral neck (g/cm^3^), mean (SD)		0.61 (0.06)	0.59 (0.06)	.27	0.303
BMD lumbar spine (g/cm^3^), mean (SD)		0.85 (0.12)	0.91 (0.16)	.17	0.391
**Smoking, n (%)**				>.99	0.005
	No	31 (91)	21 (91)		
	Yes	3 (9)	2 (9)		
**Self-sustaining, n (%)**				.74	0.103
	No	6 (18)	5 (22)		
	Yes	28 (82)	18 (78)		
**Daily leaving apartment, n (%)**				.05	0.566
	No	4 (12)	8 (35)		
	Yes	30 (88)	15 (65)		
**Weekly sports activity (>3 h), n (%)**				.06	0.569
	No	10 (29)	13 (57)		
	Yes	24 (71)	10 (43)		

^a^SMD: standardized mean difference.

^b^EQ-5D-5L: European Quality of Life 5-dimension questionnaire.

^c^SPPB: Short Physical Performance Battery.

^d^SARC-F: sarcopenia test (strength, assistance with walking, rising from a chair, climbing stairs, and fall).

^e^BMD: bone mineral density.

**Table 3:** Comparison of gait parameters between patients with and without physical frailty.

Variable	No physical frailty, mean (SD)	Physical frailty, mean (SD)	*P* value	SMD^a^
Mean gait speed (m/s)	1.09 (0.28)	0.69 (0.19)	<.001	–1.637
TUG^b^ time (s)	8.52 (1.93)	15.79 (5.50)	<.001	1.765
Mean stride length (m)	1.12 (0.19)	0.85 (0.17)	<.001	–1.450
Mean gait cadence (strides/min)	59.72 (8.83)	49.37 (8.21)	<.001	–1.214
Mean gait cycle time (s)	1.05 (0.16)	1.27 (0.20)	<.001	1.199
Mean double support time (s)	0.40 (0.13)	0.51 (0.14)	.003	0.843
Number of steps (n)	15.32 (6.05)	20.04 (5.67)	.005	0.804
Mean acceleration over gait cycle right (*g*)	0.03 (0.89)	0.59 (0.74)	.02	0.695
COP^c^ trace length right (m)	5.25 (1.96)	7.06 (3.22)	.02	0.680
Mean acceleration over gait cycle right (*g*)	–2.36 (1.32)	–1.39 (1.54)	.02	0.672
Mean length width of gait line right (mm)	131.10 (21.20)	142.66 (19.05)	.04	0.574
Variance of acceleration over gait cycle (m/s^2^)	1.66 (0.86)	1.21 (0.78)	.05	–0.552

^a^SMD: standardized mean difference.

^b^TUG: Timed-Up-and-Go.

^c^COP: center of pressure.

**Table 4:** Comparison of physical frailty prediction methods.

Performance metric	SARC-F^a^ LR^b^	TUG^c^ test LR	KNN^d^ classifier	RF^e^ classifier
Accuracy	0.684	0.667	0.719	0.724
AUROC^f^	0.639	0.862	0.919	0.859

^a^SARC-F: sarcopenia test (strength, assistance with walking, rising from a chair, climbing stairs, and fall).

^b^LR: logistic regression.

^c^TUG: Timed-Up-and-Go.

^d^KNN: K-nearest neighbor.

^e^RF: random forest.

^f^AUROC: area under the receiver operating characteristic curve.

## Discussion

### Principal Findings

Based on a sample of 57 patients and advanced statistics, this study shows that gait parameters assessed by digital insoles during the TUG test outperformed both the benchmark tests (the TUG physical assessment and SARC-F questionnaire) to identify patients with physical frailty.

Patients identified as physically frail classified by their SPPB scores (≤8) were on average 5 years older than patients that were not classified as physically frail, with no significant difference in BMI or body composition. By contrast, previous studies have reported a decreased muscle mass and increased fat percentage in patients with physical frailty [36]. Despite the considerable amount of physical frailty–related data collected (Tables 1 and 2), the vast majority (8 out of 9) of the parameters selected by the recursive feature elimination algorithm were insole gait parameters collected during the TUG test. Although the temporal gait variables such as gait speed, double support time, and gait cadence can be considered dependent variables, they all reflect different aspects of gait. For this reason, it makes sense to integrate several of these aspects into the machine learning algorithms to better map the gait pattern of an individual patient and derive the best possible classification.

Previous studies have proposed that gait speed is the most relevant parameter to identify patients with physical frailty [4]. It has been shown that a slow gait speed is associated with an increased fall risk [37], as well as a higher mortality rate [38]. Interestingly, the advanced modeling used in this study weighted stride length equally important as gait speed to differentiate between physical frailty and no physical frailty in patients, in terms of their classification importance measured by the Gini impurity (Figure 2). Although gait speed is easily assessed, it might be biased by patients’ motivation. One can hypothesize a “white coat effect,” in this case a higher level of motivation during medical gait speed examinations. Stride length might be a more robust (ie, harder to influence consciously) parameter in such settings, which might explain its superiority in the herein applied modeling. Espy et al [39] provided a possible explanation for the higher robustness of stride length compared to gait speed. They were able to show that a slow gait leads to instability, which again is compensated for by a small-stepped gait pattern [39]. It appears reasonable that patients with physical frailty would therefore compensate for their unstable gait pattern by a reduction of their stride length [39]. Overall, stride length and gait speed were found to be the two most relevant parameters for the model (Figure 2), and could only be slightly increased by adding additional gait parameters such as cadence, double support time, and acceleration over gait cycle. Consequently, stride length in addition to gait speed might be a valuable clinical parameter to identify patients with physical frailty. Their early identification is essential to reduce the number of falls [37] and possibly mortality rates [38], as well as to increase further health outcomes [40]. These considerable implications are not only important in an orthogeriatric setting but also for almost all medical specialties.

In line with previous studies*,* the SARC-F as well as the TUG test were found to be suitable for estimating the physical frailty status [41]. The slightly better results for the TUG test compared with the SARC-F might be explained by their different natures. The SARC-F is a patient-reported outcome measure, whereas the TUG test is a more objective score. Older patients have been shown to overestimate their physical abilities [42,43], which might result in false negative SARF-F scores. Complementing the SARC-F by an objective measurement such as the TUG test, handgrip strength, or a gait analysis might increase its accuracy and therefore screening value.

Nevertheless, the combination of machine learning algorithms and digital gait analysis outperformed the TUG test and SARC-F in the detection of physical frailty. The digital insoles used in this study can easily be applied and have proven to be reliable [25]. Furthermore, they could be integrated into health assessment apps, such as on a smartphone. This can facilitate both the collection of longitudinal data and remote monitoring of at-risk patients, and potentially even guide rehabilitation. Consequently, gait analysis by digital insoles might become another valuable part of the growing body of digital health devices.

### Limitations and Strengths

An obvious limitation of this study is the limited number of patients. The smaller the number of patients the algorithm is trained on, the more limited is its generalizability. Therefore, the herein proposed algorithm must be validated in a larger cohort. In the setting of a longitudinal, multicenter trial, the applied statistics could be extended to deep learning methods such as neural networks, which could further increase the accuracy of the predictions. Another limitation is the definition of physical frailty. Due to the current setup, it was only possible to define physical frailty by the SPPB. Although the SPBB is considered one of the benchmark tests for physical frailty [44], it would be even more meaningful to directly assess the occurrence of various health impairments such as falls, fractures, progression to impaired ambulation, or death. Nonetheless, these parameters can only be assessed in a longitudinal study setup.

Despite these limitations, several strengths of this study are noteworthy. First, the combined use of modern wearables and data analysis strategies from the field of data science to complement the classic statistical analysis is an advantage of this study. Due to the increasing amount of data points collected by digital devices, advanced statistics will become the primary working horse to analyze the data. Second, the meta-modeling approach applied represents a pessimistic estimation of the models’ performance in a larger cohort. Nevertheless, the resulting AUROC values of 0.801 and 0.841 can be judged as excellent [45]. These excellent results argue for the value of digital insole gait parameters. For application in clinical practice, it is conceivable that a doctor will receive an analysis on their terminal device in real time during the test, which can provide time-efficient support in clinical decision-making for or against prescribing fall prevention training, certain medications, or other therapeutic interventions. Finally, this study also indicates that gait parameters might be a promising target for physical frailty therapies. It can by hypothesized that focused physiotherapy or fall risk minimization counseling could counteract physical frailty and thereby increase the patient’s health-related quality of life.

### Conclusion

Machine learning algorithms–based gait analysis using mobile insoles appears to be a promising approach to screen for physical frailty in an outpatient setting. Due to the increasing amount of data collected, high-performance data processing will become increasingly important. Future large-scale, longitudinal, and multicenter screening trials should collect as many data points as possible, including from digital devices such as wearables, and apply advanced statistics to increase the diagnostic sensitivity and accuracy of physical frailty diagnosis.

## Abbreviations

AUROC: area under the receiver operating characteristic curve
EQ-5D-5L: European Quality of Life 5-dimension
KNN: K-nearest neighbor
RF: random forest
ROC: receiver operating characteristic
SARC-F: sarcopenia questionnaire (strength, assistance with walking, rising from a chair, climbing stairs, and falls)
SPPB: Short Physical Performance Battery
TUG: Timed Up and Go
